# Fretting and Fretting Corrosion Behavior of Additively Manufactured Ti-6Al-4V and Ti-Nb-Zr Alloys in Air and Physiological Solutions

**DOI:** 10.3390/jfb15020038

**Published:** 2024-02-05

**Authors:** Annsley O. Mace, Michael A. Kurtz, Jeremy L. Gilbert

**Affiliations:** Clemson—Medical University of South Carolina Bioengineering Program, Department of Bioengineering, Clemson University, Charleston, SC 29464, USA; annsleymace@gmail.com (A.O.M.); kurtz3@clemson.edu (M.A.K.)

**Keywords:** additive manufacturing, titanium alloys, fretting, corrosion, Ti-6Al-4V, tribocorrosion

## Abstract

Additive manufacturing (AM) of orthopedic implants has increased in recent years, providing benefits to surgeons, patients, and implant companies. Both traditional and new titanium alloys are under consideration for AM-manufactured implants. However, concerns remain about their wear and corrosion (tribocorrosion) performance. In this study, the effects of fretting corrosion were investigated on AM Ti-29Nb-21Zr (pre-alloyed and admixed) and AM Ti-6Al-4V with 1% nano yttria-stabilized zirconia (nYSZ). Low cycle (100 cycles, 3 Hz, 100 mN) fretting and fretting corrosion (potentiostatic, 0 V vs. Ag/AgCl) methods were used to compare these AM alloys to traditionally manufactured AM Ti-6Al-4V. Alloy and admixture surfaces were subjected to (1) fretting in the air (i.e., small-scale reciprocal sliding) and (2) fretting corrosion in phosphate-buffered saline (PBS) using a single diamond asperity (17 µm radius). Wear track depth measurements, fretting currents and scanning electron microscopy/energy dispersive spectroscopy (SEM/EDS) analysis of oxide debris revealed that pre-alloyed AM Ti-29Nb-21Zr generally had greater wear depths after 100 cycles (4.67 +/− 0.55 µm dry and 5.78 +/− 0.83 µm in solution) and higher fretting currents (0.58 +/− 0.07 µA). A correlation (R^2^ = 0.67) was found between wear depth and the average fretting currents with different alloys located in different regions of the relationship. No statistically significant differences were observed in wear depth between in-air and in-PBS tests. However, significantly higher amounts of oxygen (measured by oxygen weight % by EDS analysis of the debris) were embedded within the wear track for tests performed in PBS compared to air for all samples except the ad-mixed Ti-29Nb-21Zr (*p* = 0.21). For traditional and AM Ti-6Al-4V, the wear track depths (dry fretting: 2.90 +/− 0.32 µm vs. 2.51 +/− 0.51 μm, respectively; fretting corrosion: 2.09 +/− 0.59 μm vs. 1.16 +/− 0.79 μm, respectively) and fretting current measurements (0.37 +/− 0.05 μA vs. 0.34 +/− 0.05 μA, respectively) showed no significant differences. The dominant wear deformation process was plastic deformation followed by cyclic extrusion of plate-like wear debris at the end of the stroke, resulting in ribbon-like extruded material for all alloys. While previous work documented improved corrosion resistance of Ti-29Nb-21Zr in simulated inflammatory solutions over Ti-6Al-4V, this work does not show similar improvements in the relative fretting corrosion resistance of these alloys compared to Ti-6Al-4V.

## 1. Introduction

Titanium alloys are widely used in medical devices due to their enhanced fatigue strength and biocompatibility [1]. Ti-6Al-4V is particularly favored for permanently implanted medical devices because of its corrosion resistance, promoted by a thin 2–10 nm TiO_2_ passive oxide film that rapidly forms on its surface [2,3,4,5].

At the biology–device interface in vivo, tribocorrosion may temporarily disrupt the oxide film, exposing the underlying metal to the surrounding solution and causing accelerated corrosion (i.e., tribocorrosion or mechanically assisted corrosion) [6,7,8,9,10,11,12]. Documented in vivo damage modes include fretting, wear, pitting, hydrogen embrittlement and selective dissolution of the Ti-6Al-4V β phase [13,14,15]. During fretting crevice corrosion, wear is hypothesized to be an essential component of the mechanism [16,17,18]. Retrieval studies of monobloc Ti-6Al-4V femoral stems show tribocorrosion on Ti-6Al-4V femoral heads, promoted by bearing wear mechanics [19,20]. Additionally, previous in vitro research establishes Ti-6Al-4V as a poor bearing surface [21,22], leading to in vivo failure [19,23,24,25,26,27,28]. Thus, wear resistance, fretting, and tribocorrosion properties are important considerations for new titanium biomaterials.

In response to aluminum and vanadium toxicity concerns, alternative titanium-based alloys of the Ti-Nb-Zr ternary system have been introduced [29,30]. Niobium and zirconium have historically been used as β and neutral stabilizing elements, respectively, for titanium alloys. Compared to Ti-6Al-4V, Ti-13Nb-13Zr shows superior corrosion resistance in inflammatory simulating solutions [31,32,33]. While the addition of H_2_O_2_ alters the structure of the oxide film for both alloys, increasing thickness and defects, the oxide polarization resistance for Ti-6Al-4V rapidly degrades under less in vitro exposure time [33,34]. Compared to Ti-6Al-4V, Ti-13Nb-13Zr exhibits decreased wear properties [35,36]. Tin (Sn) may be further added to Ti-Nb-Zr alloys to improve their super-elastic capabilities while maintaining a low modulus [37,38].

AM Ti-6Al-4V devices are implanted with increasing frequency [39,40]. Unlike traditional manufacturing, AM allows for the development of complex and highly tunable geometries. In vivo, AM acetabular components promote bone ingrowth at the device interface with a modulus and structure mimicking native bone, a favorable characteristic when osseointegration and stress shielding are concerns [41,42,43,44,45]. AM also produces customizable implants for patients who may exhibit substantial bone volume loss or poor bone density, prohibiting the use of off-the-shelf modular orthopedic devices. Finally, metallurgists use AM to rapidly develop and test new titanium alloys and admixtures that may not otherwise be manufactured using traditional methods. It is important to note here that the well-established bi-modal equiaxed α + β microstructure used in traditional Ti-6Al-4V alloys arising from thermomechanical processing and mill annealing is not developed in the rapid melt and re-solidification processes of AM. Despite the alterations in microstructure, the clinical results for AM Ti-6Al-4V devices are generally positive, though the fretting corrosion properties remain poorly understood [46,47,48].

Previous work from our group investigated the fundamental corrosion properties of three new AM biomaterials: (1) Ti-6Al-4V with 1% added nano yttria stabilized ZrO_2_ particles (1% nYSZ); (2) admixed Ti-29Nb-21Zr and (3) pre-alloyed Ti-29Nb-21Zr [49]. In a simulated inflammatory environment, pre-alloyed and admixed Ti-29Nb-21Zr passivation current densities and impedance magnitudes remained unaffected by the addition of H_2_O_2_, while Ti-6Al-4V corrosion properties decreased. These results indicate that AM Ti-29Nb-21Zr and Ti-6Al-4V 1% nYSZ may be suitable for in vivo use, meriting investigation into their fretting corrosion properties.

While Ti-29Nb-21Zr shows improved corrosion resistance, a gap exists in our understanding of the alloy’s wear and fretting corrosion properties. In this study, we investigated the wear and fretting corrosion properties of Ti-6Al-4V 1% nYSZ, admixed Ti-29Nb-21Zr and pre-alloyed Ti-29Nb-21Zr, using traditionally manufactured Ti-6Al-4V and AM Ti-6Al-4V as comparisons [50,51,52]. We used a 17 µm radius diamond asperity under fixed loads and sliding distances, allowing for a precise, controlled, and repeatable method to generate quantifiable damage [53]. Post-test analysis of the wear depth, the damage modes and the local oxidation behavior were also investigated. Based on the results of previous corrosion studies on conventionally manufactured alloys, where Ti-13Nb-13Zr showed increased corrosion resistance in inflammatory simulating solutions and decreased wear properties versus Ti-6Al-4V, we hypothesize a similar relationship here with AM Ti-29Nb-21Zr and AM Ti-6AL-4V [31,32,35,36]. Additionally, from the positive clinical results of AM Ti-6Al-4V devices, we expect similar wear properties between the Ti-6Al-4V-based biomaterials irrespective of the underlying microstructural differences between conventional and AM alloys [46,47,48].

## 2. Materials and Methods

Ti-6Al-4V extra low interstitial (ELI, ASTM F-136), AM Ti-6Al-4V, AM Ti-6Al-4V + 1% nYSZ, AM Ti-29Nb-21Zr (admixture), and AM Ti-29Nb-21Zr (pre-alloyed) were chosen for testing. The as-printed samples were provided by Z3Dlabs SAS, Paris, France.

### 2.1. AM Titanium Printing Parameters

AM titanium discs were designed and printed using laser powder bed fusion (L-PBF) on an SLM 125 HL printer (SLM Solutions Group AG, Lubeck, Germany). Printer parameters are listed in Table 1. Parameters were previously optimized for Ti-6Al-4V 1% nYSZ mechanical properties and density [50]. Characterization of the precursor powders for the five materials used in this study is reported by Kurtz et al. [49].

Following L-PBF, the alloy samples were thermally annealed for 3 h at 600 °C followed by air cooling.

### 2.2. Microstructure

Alloy surfaces were polished with a 0.3 µm alumina suspension, rinsed with deionized (DI) water and 70% ethanol, and polished with 60% colloidal silica and 40% 9.8 M H_2_O_2_. Backscattered (BSE) and secondary electron (SE) micrographs were captured using scanning electron microscopy (SEM, Hitachi S-3700N, Tokyo, Japan). Accelerating voltages ranging from 10–15 kV were used. After imaging, the discs were repolished to 0.3 µm and cleaned with DI water.

### 2.3. Dry Fretting—Tribology

**Figure 1:**
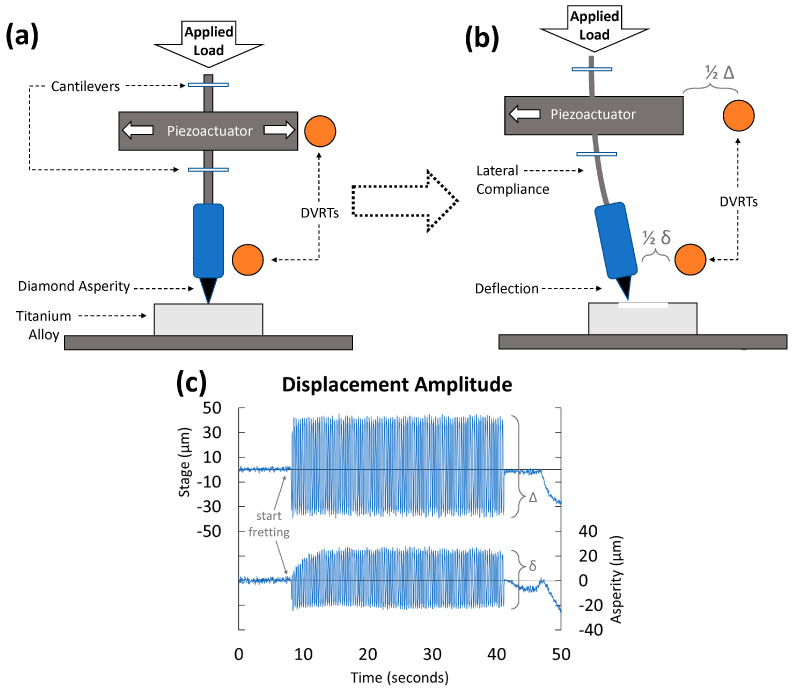
(**a**) Diagram of fretting apparatus [54]. (**b**) System after applied horizontal movement (Δ) showing lateral compliance and asperity movement (δ), with a difference of deflection (Δ − δ = d) of the system under applied load (F_N_) and dependent on COF. As denoted in the figure, lateral compliance refers to the stiffness of the vertical system (i.e., its resistance to bending under horizontal (perpendicular) loads), and deflection refers to the amount of one end of the vertical system (the asperity at the base) is diverted from its neutral point (i.e., the asperity location when 0 horizontal load is applied). (**c**) Example data set of DVRT (differential variable reluctance transducers) displacement measurements of the applied and asperity amplitude over 100 fretting cycles (at 3 Hz).

A previously custom-built micro-fretting apparatus was used [53,54] consisting of a single 17 µm radius diamond asperity. A micromotor controlled vertical movement, while horizontal movements (wear) were performed by a piezoelectric actuator (Modular Piezo Controller System ENV, Piezosystem Jena, Inc., Hopedale, MA, USA) and a function generator (SFG-210, Global Specialties, Yorba Linda, CA, USA) (Figure 1).

With no solution present (dry wear), cyclic fretting tests were performed for 100 cycles at 3 Hz (33 s of fretting) under a constant load of 100 mN of normal force (F_N_) applied with DC motors. Using Hertzian contact mechanics, this corresponds to 6.2 GPa nominal contact stress, indicating significant plasticity in the contact region [53]. Horizontal motion was applied in a square wave function. For each trial, fretting was initiated at 7 s and ended at 40 s of a 50 s test. The time before and after fretting measured the non-fretting baseline currents from which the fretting currents could be determined. After testing, wear tracks were nominally 80 +/− 10 µm long, measured with a digital optical microscope (DOM, Keyence VHX-6000, Mahwah, NJ, USA).

In some cases, the true scratch was shorter due to the higher coefficient of friction (COF) and lateral compliance of the load system. Two differential variable reluctance transducers (DVRT) were mounted on the system to monitor the asperity’s horizontal micromotion and the true horizontal amplitude. Fretting amplitude was recorded with a data acquisition system (LabVIEW, National Instruments, Austin, TX, USA). Testing was repeated on each alloy multiple times (n = 5).

### 2.4. Fretting in Solution—Fretting Corrosion

Open circuit potential (OCP vs. Ag Ag/Cl) was monitored for one minute before applying a potentiostatic hold (0 V vs. Ag/AgCl), and subsequent baseline current plateaued before fretting corrosion testing in phosphate-buffered saline (PBS, ASTM F2129 Table X2.3, P3813-10PAK, Sigma-Aldrich, St. Louis, MO, USA). A three-electrode potentiostat (EG&G 263A, Princeton Applied Research/Ametek, Inc., Berwyn, PA, USA) was used for electrochemical measurements with a carbon counter electrode, a chlorinated silver wire reference electrode, and the titanium biomaterials as working electrodes. Fretting currents were recorded with the same LabVIEW data acquisition system used for DVRT measurements.

Fretting corrosion was repeated in solution under the same conditions (100 mN, 3 Hz, 100 cycles) (n = 5). Surface damage was analyzed with DOM and SEM.

### 2.5. Post-Test Imaging and Analysis

#### 2.5.1. Depth of Wear

**Figure 2:**
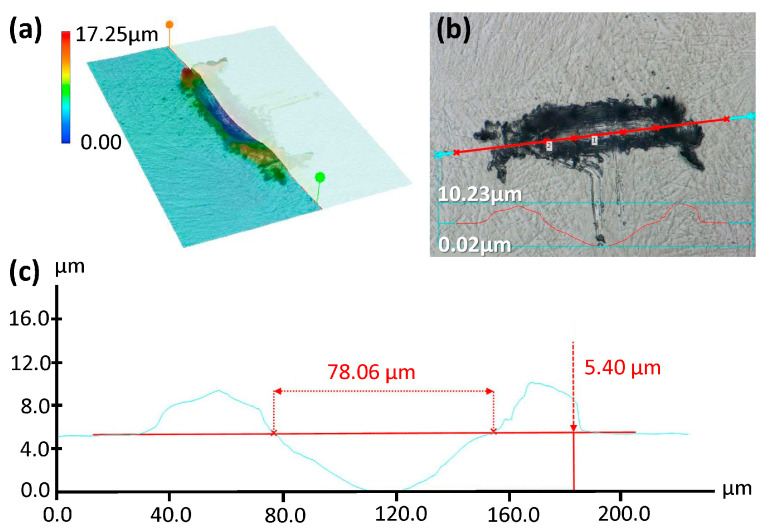
(**a**) A representative DOM 3D reconstruction and heat map of wear track damage and debris pile-up after 100 cycles of uniaxial fretting corrosion. Conditions included a 100 mN normal load, a 3 Hz frequency, and a 17 µm radius diamond asperity on pre-alloyed AM Ti-29Nb-21Zr in PBS. The heat map scale bar corresponds with increases in depth in the + Z direction (assuming a three-dimensional Euclidian space). Here, the bottom of the wear track represents the global minimum (blue, 0 µm), and the peak of the debris pile-up represents the global maximum (red, 17.25 µm). (**b**) A 2D surface image shows the line scan location and (**c**) depth and length measurement from that line scan. The horizontal red line in (**c**) corresponds with the sample surface while the blue line follows the debris pileup and subsurface wear track. Axes and micron markers are superimposed on the original DOM images to improve legibility.

Wear track length was measured using DOM. Images were captured in 3D mode to quantify the depth. Using Depth from Defocus (DFD) 2018 software, 3D reconstructions of the damaged regions were analyzed. Tribocorrosion (in PBS) damage on pre-alloyed Ti-29Nb-21Zr is shown in Figure 2 as an example [53].

Depth measurements were compared between dry fretting and fretting corrosion groups and between the five titanium biomaterials.

#### 2.5.2. Electrochemical Measurements

**Figure 3:**
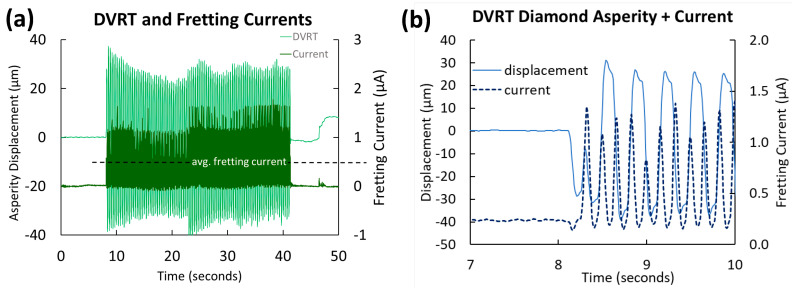
Horizontal asperity displacement (light) and fretting current above baseline (dark) measurements before, during, and after uniaxial fretting corrosion (**a**) for 100 cycles with an applied 100 mN normal load at 3 Hz on admixed AM Ti-29Nb-21Zr (17 µm radius diamond asperity, 0 V vs. Ag/AgCl in PBS). Note the non-uniform fretting amplitude of the asperity and spikes in current correlating to sticking/slipping of the asperity during fretting. (**b**) DVRT and current were recorded during the start of fretting, just after 8 s, showing 11 spikes in current for 5.5 cycles of loading (3 Hz movement back and forth).

Fretting currents were recorded using LabVIEW 2023 Q3 software in PBS for each alloy and were plotted versus time (Figure 3).

Both the fretting current (the average current above baseline over the 33 s of fretting) and baseline current were compared between the five titanium biomaterials.

#### 2.5.3. Elemental Analysis of Debris

SEM was used to image wear tracks, documenting the local damage, plastic deformation mechanisms, and the debris field with both secondary (SE, topographical) and backscattered (BSE, chemistry) electron imaging modes.

Debris pileup and embedding within the wear scratch were chemically analyzed using energy dispersive spectroscopy (EDS, Aztec, Oxford Instruments, Abingdon, UK). Using Aztec Version 4.0 software, elemental weight percent measurements were acquired for Ti, Al, V, Zr, O, and P. Quantities of each element were compared between Ti-6Al-4V, AM Ti-6Al-4V, and AM Ti-6Al-4V 1% nYSZ, as well as between both AM Ti-29Nb-21Zr samples. Oxide debris produced during dry fretting and fretting corrosion was also quantified for all five biomaterials.

### 2.6. Statistical Analysis

Statistical analysis was conducted with analysis of variance (ANOVA) methods, implementing Bonferroni corrections or Tukey’s post-hoc comparisons where appropriate (α = 0.05). Depending on the variable in question, either single-factor or two-way analyses were performed with a sample size of n = 5 for each group.

## 3. Results

### 3.1. Microscopy

#### 3.1.1. Microstructure

**Figure 4:**
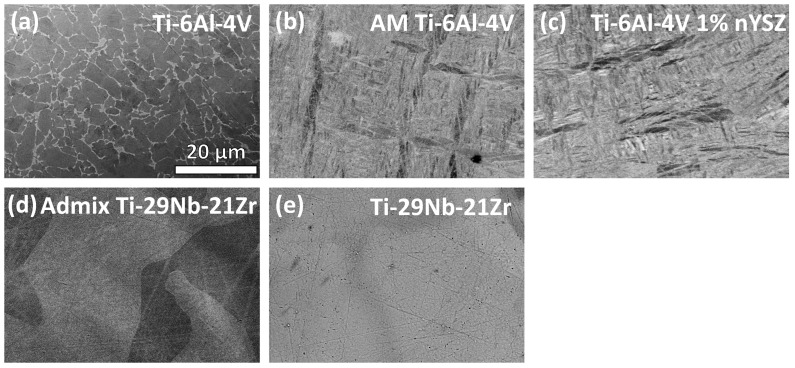
SEM backscatter micrographs (×2000 magnification) of (**a**) traditional Ti-6Al-4V, (**b**) AM Ti-6Al-4V, (**c**) AM Ti-6Al-4V 1% nYSZ, (**d**) admixed AM Ti-29Nb-21Zr, and (**e**) pre-alloyed AM Ti-29Nb-21Zr [49]. Note the equiaxed two-phase microstructure for (**a**) traditional Ti-6Al-4V, the martensitic microstructures for (**b**) AM Ti-6Al-4V and (**c**) AM Ti-6Al-4V %1 nYSZ, and the heterogenous microstructure of (**d**) the admixed Ti-29Nb-21Zr, where the differently contrasting regions represent localized melt-pools of Ti, Nb, and Zr. No visible microstructure is present for (**e**) the pre-alloyed AM Ti-29Nb-21Zr. (Note: As (**b**–**e**) are of the same scale as (**a**), the scalebar in (**a**) applies to all micrographs in Figure 4).

Polished alloy surfaces were viewed under SEM (Figure 4).

The SEM BSE micrograph of traditional Ti-6Al-4V in Figure 4a shows a two-phase equiaxed microstructure. The vanadium-rich β phase appears distinctly brighter and takes up less surface area than the darker, globular, aluminum-rich α phase. Both AM Ti-6Al-4V (Figure 4b) and Ti-6Al-4V 1% nYSZ (Figure 4c) have martensitic microstructures. Note the needle-like appearance, characteristic of the SLM powder bed fusion manufacturing process for α and α + β alloys. Both alloys (Figure 4b,c) have lamellar α grains approximately 20 μm in length that appear darker gray than the lighter and smaller martensitic needles in the rest of the micrograph. The admixed Ti-29Nb-21Zr microstructure shown in Figure 4d is comprised of several distinct regions of varying contrast, corresponding to the rapid melting and re-solidification of the admixed Ti, Nb, and Zr powder particles. The EDS of the precursor powder for the admixed Ti-29Nb-21Zr (not shown here) reveals a mixture of three separate powders (Ti, Nb, and Zr), including spherical Ti and Zr particles and blocky Nb particles [49]. No discernable microstructure is revealed from the mechanical polishing of the pre-alloyed Ti-29-Nb-21Zr, as shown in Figure 4e. 

#### 3.1.2. Wear Track Damage and Debris

**Figure 5:**
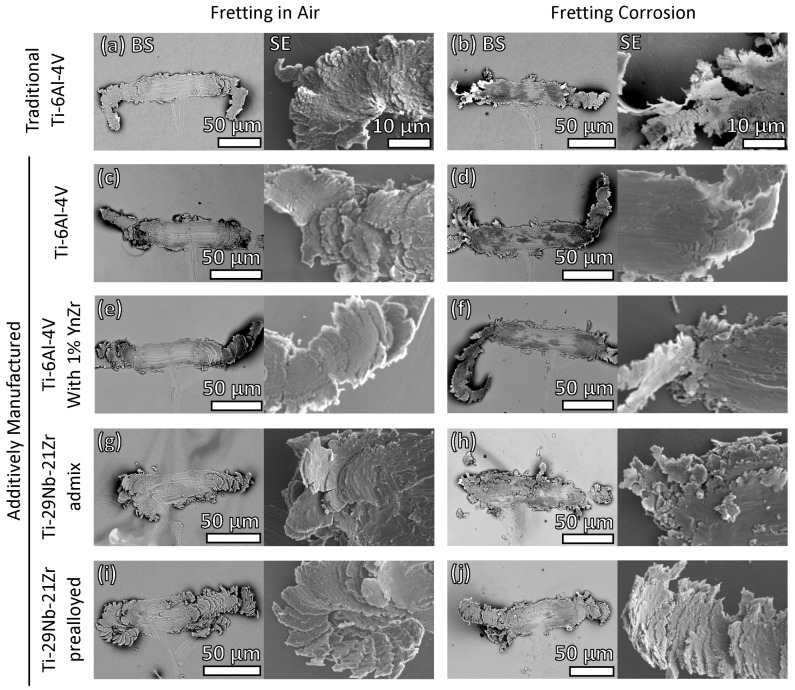
Paired backscatter (BSE) and secondary (SE) micrographs for the five tested biomaterials in air (**a**,**c**,**e**,**g**,**i**) and in PBS (**b**,**d**,**f**,**h**,**j**). The SEM BSE micrographs (left, between ×650 and ×850 magnification) show the entire damaged region and debris field. The higher magnification SE micrographs (right, ×3000 magnification) show the nature of the wear track and debris removal in dry conditions versus in PBS. Note the increase in dark regions in the micrographs captured after fretting corrosion, indicative of increased oxide generation in the wear tracks. (Note: All SE images (right, ×3000) scales are equivalent. Thus, the scale bars shown in (**a**,**b**) apply to all SE micrographs.

SEM BSE analysis of the resulting wear tracks and SE micrographs of the debris document the nature of damage between alloys as well as between fretting in air and fretting corrosion in PBS (Figure 5).

Figure 5 shows a comparison of surface damage after fretting in air (Figure 5a,c,e,g,i) and in PBS (Figure 5b,d,f,h,j) under a passive potentiostatic hold (0 V vs. Ag Ag/Cl) for traditional Ti-6Al-4V (Figure 5a,b), AM Ti-6Al-4V (Figure 5c,d), AM Ti-6Al-4V 1% nYSZ (Figure 5e,f), admixed AM Ti-29Nb-21Zr (Figure 5g,h), and pre-alloyed AM Ti-29Nb-21Zr (Figure 5i,j). There is evidence of cyclic plastic deformation, plate-like particle formation, oxidation, ribboning, removal of metal, and oxide/debris impaction into the wear track for each alloy in both dry and wet conditions. The BSE images of damage in the air show smaller amounts of oxide (dark regions) embedded into the track compared to the images taken after fretting in solution. All five titanium metals show no clear evidence of slip lines, and material removal is primarily by cyclic shearing, cutting and plowing of the diamond tip, which results in ribbons of debris forming at each end of the sliding stroke.

The most visibly notable difference in damage generated between dry fretting (Figure 5a) and fretting corrosion (Figure 5b) of traditional Ti-6Al-4V appears to be additional oxide embedded into the wear track during fretting in PBS. Additionally, the wear mechanism is affected by the presence of the solution. During dry fretting, one large ribbon is typically extruded (Figure 5a) compared with multiple smaller ribbons generated in solution (Figure 5b). Similarly, more oxide is embedded in PBS (Figure 5d,f) than in air (Figure 5c,e) for AM Ti-6Al-4V and AM Ti-6Al-4V 1% nYSZ. Fretting corrosion results in larger extrusion ribbons and debris for both AM Ti-6Al-4V (Figure 5d) and AM Ti-6Al-4V 1% nYSZ (Figure 5f) compared to dry fretting (Figure 5c,e).

During dry fretting, the asperity follows a straight wear track when abrading the traditional and AM Ti-6Al-4V alloys (Figure 5a,c,e), plowing through and displacing the alloy uniformly. The Ti-29Nb-21Zr alloy grains were not as easily displaced, causing the asperity to slide in a non-linear fashion (Figure 5g,i). The BSE images in Figure 5g,h show the heterogeneous chemistry of the microstructure of admixed AM Ti-29Nb-21Zr, inducing asperity wandering during fretting. This damage is influenced by the location and orientation of the variable chemistry associated with admixed elements and their rapid solidification. However, more oxide debris appears in the AM Ti-29Nb-21Zr (admixed) fretting corrosion wear track (Figure 5h) than in the dry fretting track (Figure 5g). Additionally, more loose debris dislodged, generating a debris field around the asperity path, separate from the pileup on the ends of the path. Compared to the traditional and AM Ti-6Al-4V biomaterials (Figure 5a–f), both AM Ti-29Nb-21Zr materials (Figure 5g–j) BSE micrographs show evidence of asperity sticking, indicated by a shorter wear track and more debris pileup within the track.

#### 3.1.3. Elemental Analysis of Debris

**Figure 6:**
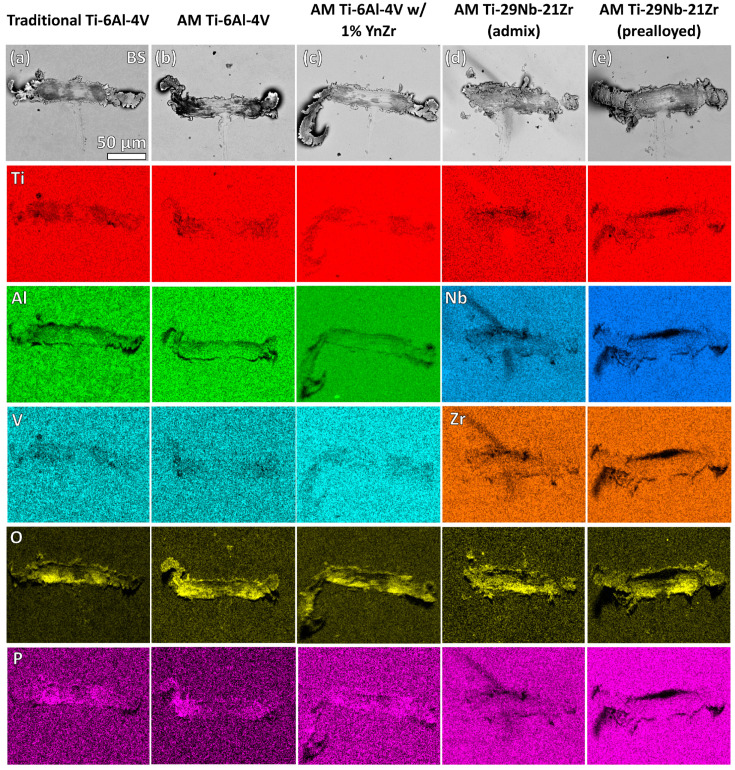
Wear track imparted by a single diamond asperity fretting corrosion apparatus in PBS on (**a**) traditional Ti-6Al-4V, (**b**) AM Ti-6Al-4V, (**c**) AM Ti-6Al-4V 1% nYSZ, (**d**) admixed AM Ti-29Nb-21Zr, and (**e**) pre-alloyed AM Ti-29Nb-21Zr. SEM SE micrographs show the nature of the surface damage as well as the debris pile-up. False color EDS maps identify elemental mapping of titanium, aluminum, niobium, zirconium, phosphorous, and oxygen. (Note: All images in (**a**–**e**), regardless of the element shown, are of the same magnitude as (**a**). Thus, the scale bars shown in (**a**) apply to all images in Figure 6).

EDS analysis performed on the damaged region and surrounding debris field after fretting corrosion damage for relevant elements is shown below in Figure 6.

Analysis of traditional Ti-6Al-4V (Figure 6a), AM Ti-6Al-4V (Figure 6b), AM Ti-6Al-4V 1% nYSZ (Figure 6c) and the pre-alloyed AM Ti-29Nb-21Zr (Figure 6e) reveals a homogenous distribution of titanium (red), aluminum (green, Ti-6Al-4V), niobium (blue, Ti-29Nb-21Zr), and zirconium (orange, Ti-29Nb-21Zr). Conversely, the admixed AM Ti-29Nb-21Zr (Figure 6d) has a heterogeneous distribution of titanium (red), niobium (blue), and zirconium (orange). Analysis of admixed AM Ti-29Nb-21Zr (Figure 6d) shows that areas higher in titanium correspond with lower counts of niobium and zirconium. All five titanium biomaterials feature elevated levels of oxygen (yellow) within the wear track and the surrounding debris. Ti-6Al-4V-based biomaterials (Figure 6a–c) have elevated levels of phosphorous (pink, likely from phosphates) within the wear track and debris compared to the surrounding surface. Additional EDS analysis (not shown) indicates zirconium phosphates, not niobium, present on admixed AM Ti-29Nb-21Zr (Figure 6d).

**Figure 7:**
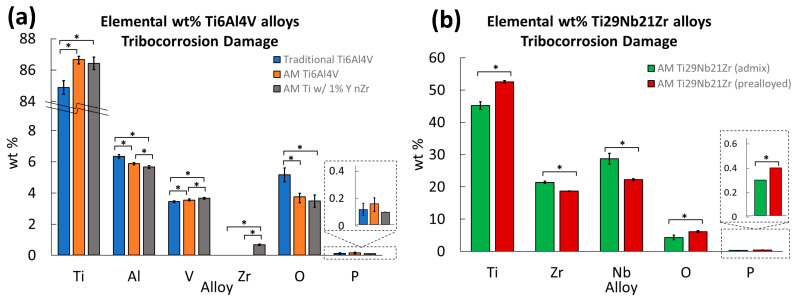
Average EDS weight percent of elements Ti, Al, V, Zr, and Nb, as well as O and P (found in oxides and phosphates) for (**a**) traditional Ti-6Al-4V, AM Ti-6Al-4V, and AM Ti-6Al-4V 1% nYSZ and (**b**) admixed and pre-alloyed AM Ti-29Nb-21Zr measured on the damaged regions and debris field generated from fretting in PBS under a passive 0 V vs. Ag Ag/Cl hold, as well as comparisons between groups, n = 5. The asterisk (*) between groups indicates significance (*p* < 0.025).

EDS chemical weight percent analysis of Ti-6Al-4V (Figure 7a) and Ti-29Nb-21Zr (Figure 7b) was performed on the damaged regions after 100 cycles of fretting in solution and compared within groups.

Average values plotted in Figure 7 are reported in Table 2, along with standard deviation and statistical comparison between groups.

Table 2 lists *p*-values from a single factor ANOVA performed between traditional Ti-6Al-4V, AM Ti-6Al-4V, and AM Ti-6Al-4V 1% nYSZ showing significant differences in Ti, Al, V, and Zr, as well as weight percent of oxygen (with a Bonferroni correction factor of 2) in the debris field.

Tukey’s post-hoc between groups shows traditional Ti-6Al-4V has significantly lower counts of titanium (*p* < 0.001) and vanadium (p_Traditional vs. AM Ti-6Al-4V_ = 0.03 and p_Traditional vs. AM Ti-6Al-4V 1% nYSZ_ < 0.001), higher counts of aluminum (*p* < 0.001), and, using the weight percent of oxygen on the damaged region and debris field as a measure of oxidation, more oxidation (*p* < 0.001) than both AM Ti-6Al-4V biomaterials (Figure 7).

Similarly, in Figure 7, AM Ti-6Al-4V 1% nYSZ has significantly lower counts of aluminum (*p* = 0.01) and higher counts of vanadium (*p* = 0.01) than AM Ti-6Al-4V and higher zirconium (*p* < 0.001) than traditional or AM Ti-6Al-4V. There was no difference between AM Ti-6Al-4V and AM Ti-6Al-4V 1% nYSZ otherwise (*p* > 0.54).

When comparing elemental weight percentages between the versions of AM Ti-29Nb-21Zr, the admixed had significantly less titanium but more zirconium and niobium. The pre-alloyed version had more oxidation debris and more phosphorous (likely from the formation of phosphates, PO_4_).

**Figure 8:**
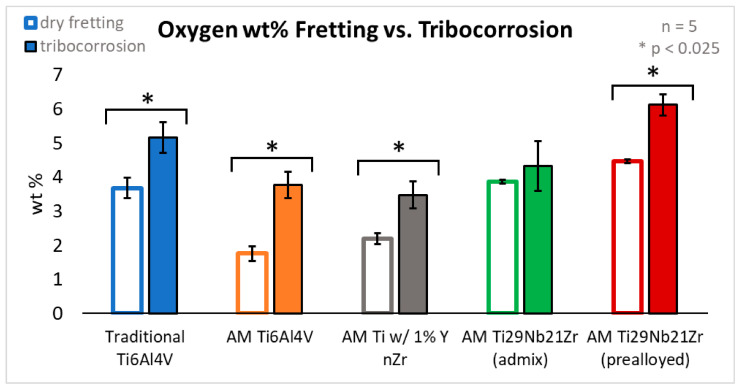
Average oxygen weight percent measured with EDS analysis of the wear track and surrounding debris with comparisons between dry fretting and fretting in PBS, n = 5. Outlined bars represent measurements from fretting wear tracks in air while solid bars indicate measurements acquired from fretting corrosion wear tracks in PBS. Colors correspond with each of the five biomaterials tested: traditional Ti-6Al-4V (blue); AM Ti-6Al-4V (orange); AM Ti-6Al-4V 1% nYSZ (grey); admixed AM Ti-29Nb-21Zr (green) and pre-alloyed AM Ti-29Nb-21Zr (red). The asterisk (*) between groups indicates significance (*p* < 0.025).

Figure 8 shows the differences, or lack thereof, in the weight percent of oxygen in the debris field and wear track generated from dry fretting to fretting corrosion.

Again, using weight percent of oxygen as a measure of oxidation, paired two-tailed Student’s *t*-tests with a Bonferroni correction factor of 2 (α = 0.025) yields significantly more oxide debris generated during fretting corrosion than during fretting. This is true for traditional Ti-6Al-4V (*p* = 0.001), AM Ti-6Al-4V (*p* = 0.001), AM Ti-6Al-4V 1% nYSZ (*p* = 0.002), and pre-alloyed AM Ti-29Nb-21Zr (*p* < 0.001), but there was no difference for admixed AM Ti-29Nb-21Zr (*p* = 0.21).

### 3.2. Extent of Wear

#### 3.2.1. Depth: DOM

**Figure 9:**
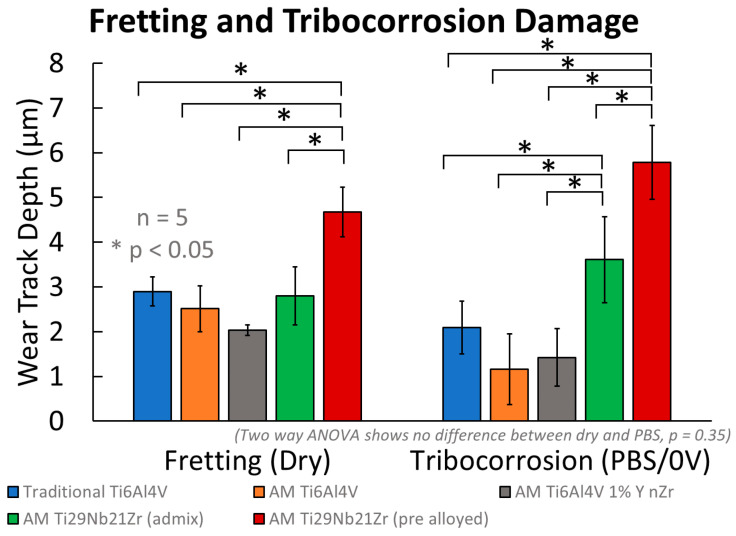
Abrasion depth from a single 17 µm radius diamond asperity after 100 cycles of fretting in air and fretting corrosion in PBS under a constant 0 V vs. Ag Ag/Cl hold. Values are reported for traditional and additively manufactured Ti-6Al-4V and Ti-29Nb-21Zr (admixed and pre-alloyed) titanium biomaterials.

Adding corrosion to the process of wear did not affect the magnitude of the wear track depth. However, fretting and fretting corrosion damage was more extensive on AM Ti-29Nb-21Zr (admixed) than any other biomaterial tested under the same conditions (Figure 9).

Two-way ANOVA (air/solution, sample) reveals no difference in the depth abraded during dry fretting and fretting corrosion (*p* = 0.35). However, the five biomaterials differed in wear track depth (*p* < 0.001). Table 3 shows average and standard deviation values (in µm) of depth abraded by a single diamond asperity after 100 cycles in air and PBS.

Tukey’s post-hoc analysis between the materials identified pre-alloyed AM Ti-29Nb-21Zr as less resistant to physical wear during dry fretting (deeper wear track) than traditional Ti-6Al-4V (*p* = 0.001), AM Ti-6Al-4V (*p* < 0.001), AM Ti-6Al-4V 1% nYSZ (*p* < 0.001), and the admixture AM Ti-29Nb-21Zr (*p* < 0.001). There were no differences between the others (*p* > 0.23). Damage from fretting corrosion, similarly, was worse on the pre-alloyed AM Ti-29Nb-21Zr than traditional Ti-6Al-4V, AM Ti-6Al-4V, AM Ti-6Al-4V 1% nYSZ, and the admixed AM Ti-29Nb-21Zr (*p* < 0.001 for each). However, the admixed AM Ti-29Nb-21Zr also experienced higher amounts of damage than traditional Ti-6Al-4V (*p* = 0.01), AM Ti-6Al-4V (*p* < 0.001), and AM Ti-6Al-4V 1% nYSZ (*p* < 0.001). No other material tested experienced different amounts of fretting corrosion damage in PBS under 0 V vs. Ag Ag/Cl (*p* > 0.17).

#### 3.2.2. Friction and Sliding Amplitude

**Figure 10:**
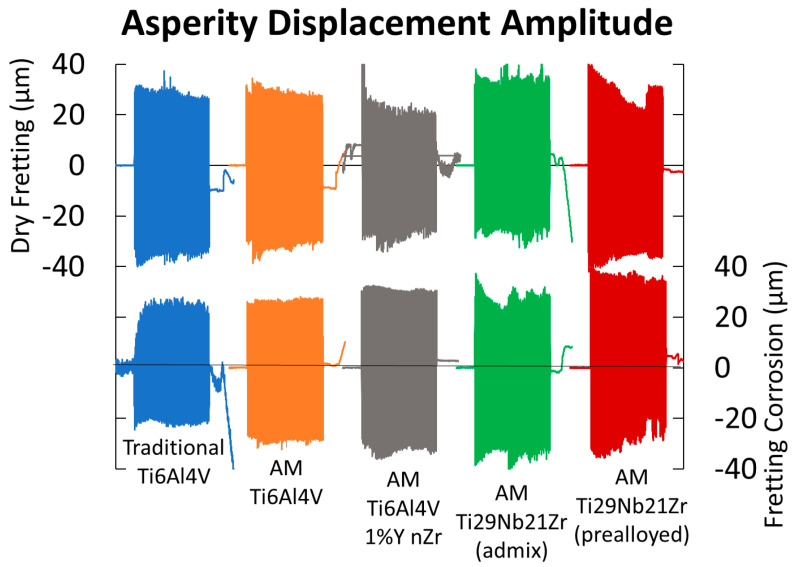
Sample fretting (top) and fretting corrosion (bottom) amplitude data (n = 1) tracked by DVRT of the diamond asperity on traditional Ti-6Al-4V, AM Ti-6Al-4V, AM Ti-6Al-4V 1% nYSZ over 100 cycles under 50 mN load. Note: Stage motion is not shown, only asperity DVRT tracking.

Figure 10 shows the effect of adding solution to fretting in terms of micromotion and the amount of sticking when horizontal motion of the fretting asperity is tracked by DVRT.

DVRT motion tracking of the asperity shows an overall difference between dry fretting and fretting in solution. Though the asperity is under a controlled amplitude input, how it moves on the surface in contact results from the frictional forces between the asperity and the metal. The asperity amplitude in solution is much more uniform overall, whereas when dry fretting and frictional forces are higher, the asperity experiences sticking more often (most prevalent for AM Ti-6Al-4V 1% nYSZ, AM Ti-29Nb-21Zr (admixed), and AM Ti-29Nb-21Zr (pre-alloyed)). This is shown by the drop in fretting amplitude after a few cycles during dry fretting, likely due to the added effects of lubrication as well as a thicker titanium oxide layer in solution compared to in air. Similarly, both AM Ti-29Nb-21Zr metals also experience asperity sticking during fretting corrosion more often than the three other metals tested. DVRT motion tracking of the stage, i.e., the controlled horizontal amplitude output (not shown in Figure 10), indicates a uniform motion of the stage (represented in Figure 1c). Thus, any difference in the horizontal motion of the asperity over time and between dry/wet fretting is due to surface forces alone.

#### 3.2.3. Electrochemical Measurements

**Figure 11:**
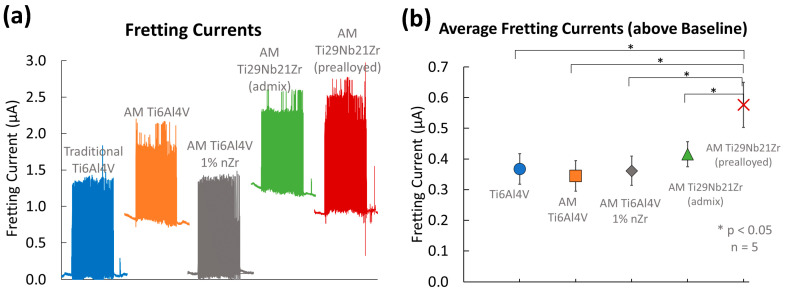
(**a**) Representative data (n = 1) of fretting and baseline current data vs. time recorded during fretting in PBS under a 0 V vs. Ag Ag/Cl potentiostatic hold concurrent with a static 100 mN load at 3 Hz. (**b**) Average fretting current above baseline for each biomaterial. Error bars represent the standard deviation (n = 5).

An example (n = 1) of baseline current before, fretting current during, and return to baseline current after stopping fretting for each biomaterial is shown in Figure 11a (PBS, 0 V vs. Ag Ag/Cl). Figure 11b shows the average fretting current over 100 cycles (with the baseline current subtracted) on each of the biomaterials. Error bars represent the standard deviation for n = 5 tests performed.

Statistical analysis with a single factor ANOVA (*p*-values reported in Table 4) reveals significant differences between baseline and fretting currents (above baseline) between the five biomaterials tested.

Post-hoc analysis identified a lower baseline current for traditional Ti-6Al-4V when compared with AM Ti-6Al-4V (*p* < 0.001), admixed AM Ti-29Nb-21Zr (*p* = 0.01), and pre-alloyed AM Ti-29Nb-21Zr (*p* = 0.04). AM Ti-6Al-4V 1% nYSZ also had a significantly lower baseline current than AM Ti-6Al-4V (*p* < 0.001), admixed AM Ti-29Nb-21Zr (*p* = 0.01), and pre-alloyed AM Ti-29Nb-21Zr (*p* = 0.04). No other significant differences in baseline current were measured before fretting (*p* > 0.16).

AM Ti-29Nb-21Zr (pre-alloyed) fretting currents were significantly higher than traditional Ti-6Al-4V (*p* < 0.001), AM Ti-6Al-4V (*p* < 0.001), AM Ti-6Al-4V 1% nYSZ (*p* < 0.001), and the admixed AM Ti-29Nb-21Zr (*p* = 0.001). All others had similar fretting currents (*p* > 0.26).

**Figure 12:**
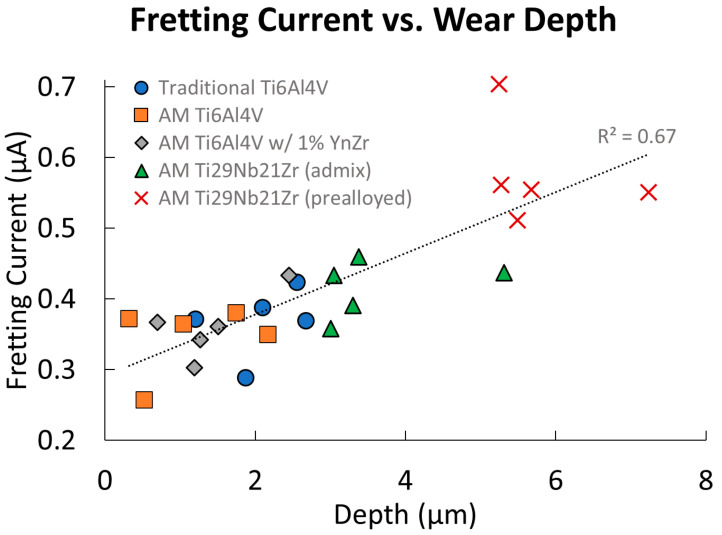
Fretting current (above baseline) versus wear track depth after 100 cycles, with each point representing one trial of one material (n = 1). Linear least squares regresses a line with R^2^ = 0.67 for all currents and depths.

Fretting current (above baseline) was averaged over 100 cycles and plotted in Figure 12 versus wear track depth. These tests were performed with matched mechanical conditions (normal force, nominal sliding distance, etc).

Fretting current (above baseline) is correlated to the depth abraded during fretting corrosion for all the titanium biomaterials (*p* < 0.001), with 67% of variation accounted for by a direct linear correlation.

## 4. Discussion

This study investigated the asperity-based fretting and fretting corrosion behavior of five titanium materials: (1) traditional Ti-6Al-4V; (2) AM Ti-6Al-4V; (3) AM Ti-6Al-4V 1% nYSZ; (4) AM Ti-29Nb-21Zr (admixed); and (5) AM Ti-29Nb-21Zr (pre-alloyed). Using a single 17 µm radius diamond stylus and reproducing an experimental setup designed by Goldberg et al., a controlled assessment of the wear and fretting corrosion resistance was performed by quantitatively measuring the damage (scratch depth and fretting currents) and qualitatively assessing debris fields and wear tracks [54].

Generally, we found few differences in wear or fretting corrosion behavior between traditional and AM Ti-6Al-4V (with and without 1% nYSZ) despite different microstructures, debris chemistries, and corrosion properties (baseline current before fretting). Additionally, the pre-alloyed AM Ti-29Nb-21Zr performed worse (i.e., greater damage, less consistent wear path, and higher fretting currents) than all three Ti-6Al-4V biomaterials tested and is also less resistant to abrasion than the admixed AM Ti-29Nb-21Zr in both air (fretting) and in solution (fretting corrosion). These findings support our initial hypotheses.

Ti-13Nb-13Zr (traditionally manufactured) data reported in the literature compare similarly to the AM Ti-29Nb-21Zr alloy and admixture results in this study. In inflammatory simulating solutions (Hanks’ solution + 0.1 M H_2_O_2_ and minimal essential media + 0.1 M H_2_O_2_), Ti-13Nb-13Zr shows improved corrosion properties compared to Ti-6Al-4V (PBS + 0.1 M H_2_O_2_) [31,32,33]. Additionally, the inferior wear resistance of the cold-rolled Ti-13Nb-13Zr alloy is well documented during block-on-disc testing (20–60 N, 0.26–1 m/s sliding speed, Ringer’s solution) [35,36]. Here, in a micro-fretting setup using a single diamond asperity (17 um radius), we show decreased wear properties for the as-built AM Ti-29Nb-21Zr alloy compared to Ti-6Al-4V biomaterials. Previously, we documented increased AM Ti-29Nb-21Zr oxide polarization resistance in PBS + 0.1 M H_2_O_2_ solution [49]. Thus, both Ti-13Nb-13Zr and AM Ti-29Nb-21Zr show improved corrosion properties and decreased wear properties when compared with Ti-6Al-4V. To enhance the wear properties of the Ti-13Nb-13Zr alloy, researchers produced a wear-resistant ceramic-oxide surface through diffusion hardening [55,56]. Applying this post-processing technique on the AM Ti-29Nb-21Zr alloy may align its fretting corrosion properties with Ti-6Al-4V, though future work is required to support this hypothesis.

To explore differences in the oxide films/fretting current magnitudes, the charge per oxide volume (*Φ* in C/cm^3^) was calculated [57].

(1) $$\Phi =\frac{\rho nF}{M_{w}}$$

where *ρ* is the oxide density, *n* is the oxide valence, *F* is Faraday’s constant (96,500 C/mol), and *M_w_* is the molecular weight of the oxide. Assuming the oxide has the same composition as the metal it is formed from (measured by EDS), the oxide charge volumes were calculated from the values listed below in Table 5.

Both versions of AM Ti-29Nb-21Zr had calculated charge volumes of approximately 15,000 C/cm^3^, which is lower than all the Ti-6Al-4V calculated values: traditional Ti-6Al-4V (20,482 C/cm^3^), AM Ti-6Al-4V (20,471 C/cm^3^), and AM Ti-6Al-4V 1% nYSZ (20,450 C/cm^3^). This is also lower than values previously calculated by Li, 2016 for traditional Ti-6Al-4V (21,498 C/cm^3^) and even lower than those reported for traditional CoCrMo (18,477 C/cm^3^) [58]. Because AM Ti-29Nb-21Zr (pre-alloyed) has a lower charge per volume of oxide abraded (C/cm^3^) yet higher fretting currents (A/s = C), this alloy experiences more oxide abrasion/repassivation than the others tested. The volume of oxide generated per charge is smaller, but the total charge generated in the same amount of time is larger; thus, more oxide volume is generated. Similarly, though the average fretting current of the admixed AM Ti-29Nb-21Zr is similar to those of the three Ti-6Al-4V biomaterials, the oxide charge volume is much smaller, meaning more oxide is abraded and repassivated to generate the same total charge during fretting.

The SEM imaging analysis of wear and fretting corrosion scars showed varying amounts of oxide debris generation and embedding within the damage zone. This result, consistent with other studies of single asperity fretting corrosion testing, decreases subsequent fretting corrosion damage and reduces the measured fretting corrosion currents [53,59]. This is, essentially, an effect arising during fretting corrosion that has only recently been identified and shows that there are antagonistic effects resulting from oxide debris embedding processes [53,59]. Additionally, EDS of the fretting corrosion debris and wear tracks (Table 2, Figure 6) reveals discrepancies in Nb and Zr content for the admixed and pre-alloyed Ti-29Nb-21Zr. These values (28.7% Nb, 21.4% Zr for the admixed and 22.3% Nb and 18.7% Zr for the pre-alloyed) were significantly different (*p* < 0.001), inconsistent with both the chemical composition of the biomaterial powders and previously measured chemical compositions of the as-built samples in the literature (30.2% Nb, 22.4% Zr for the admixed and 28.8% and 22.1% Zr for the prealloyed) [49]. One reason for this discrepancy is likely the oxidation occurring at the wear-track interface (4.32% O for the admixed and 6.12% O for the pre-alloyed) and the formation of phosphates. Chemical heterogeneities in the admixed alloy may also contribute to this measured variation. This study documents a titanium-rich melt pool approximately perpendicular to the wear track (Figure 6d). Previous studies show how the distinct elemental powder beads in the Ti-29Nb-21Zr admixture fail to fully mix during the SLM process, generating heterogenous melt pools of different chemical compositions [49]. This unique microstructure may be caused by the comparatively higher melt temperature of the cubic niobium particles, a common problem for titanium alloy and admixture powders with mismatched melt temperatures and densities [60,61].

While we investigate wear and fretting corrosion in this study, the mechanical properties of additively manufactured alloys remain critical. The fatigue resistance of AM titanium alloys is of particular concern. Voids and defects during the printing process, as well as alterations to the microstructure during the rapid heating and cooling of laser powder bed fusion, are hypothesized to decrease AM fatigue resistance. Indeed, the equiaxed α + β microstructure of traditionally manufactured Ti-6Al-4V is so widely used in medical devices because of its resistance to fatigue crack initiation. Though other microstructures (lamellar α) decrease the time needed for crack propagation, under repeated cyclic loading, preventing crack initiation takes precedence. For in vivo titanium alloy applications, including hip stems and tibial baseplates, the time and cycles needed for cracks to nucleate are exponentially larger than the time from initiation until failure. Additionally, while corrosion and wear are associated with device failure, patients may rely on a corroding device for years without experiencing clinical symptoms. In contrast, fatigue failure is catastrophic, ending in device fracture a clear breakdown of the device’s purpose [62]. Thus, crack prevention is prioritized and is a design consideration.

Here, we use as-built AM Ti-6Al-4V after a brief, stress-relieving heat treatment (600 °C for three hours). The resulting martensitic microstructure, with fine needles and prior β grains, is unlikely to reproduce the microstructure of AM Ti-6Al-4V devices used in vivo. Thus, a gap exists between the AM materials we use in this study and what is likely being implanted into patients, a limitation of our work. Overcoming this gap is nontrivial. First, device manufacturers view the post-processing they perform on AM devices as proprietary information. It is unclear what microstructures (and the processes that generate them) are present on FDA-cleared AM titanium alloy devices. Next, regulatory bodies and standards organizations lag the rapid technological developments in additive manufacturing. ASTM standards like F2924-14 *Additive Manufacturing Titanium-6 Aluminum-4 Vanadium with Powder Bed Fusion* are essential but do not specify a uniform microstructure beyond the absence of α-case, a surface layer generated under high temperatures from oxygen and nitrogen diffusion [63]. This lack of standardization may result in differing microstructures for off-the-shelf devices on a company-by-company basis. Microstructure, including the phase compositions, surface area, and elemental distribution (α is typically aluminum-rich, β vanadium-rich), influences the protective oxide film responsible for corrosion resistance and the alloys’ wear resistance, and remains a key variable for future investigations.

Conventionally and additively manufactured titanium devices in vivo are often coated or subject to post-processing and surface modifications [64]. Implant use-cases for titanium and its alloys generally avoid bearing applications. However, retrieval studies document severe corrosion in vivo on titanium alloy surfaces where rotation was not designed, including in the modular junctions of total hip and total knee replacement systems. Under cyclic loading between mixed and same-alloy titanium interfaces, mechanically assisted crevice corrosion promotes thick Cr-Ti-Mo oxides, etching, and pitting, among other damage modes [15]. Wear and fretting corrosion represent critical components during this mechanism. Asperities between two interfaces in the crevice may abrade the oxide, interrupting it and initiating a positive feedback loop that perpetuates further corrosion. In this study, we reduce this mechanism to a single diamond asperity, characterizing the fundamental wear and fretting corrosion of five titanium-based biomaterials. While these materials may not be used as rubbing surfaces, conventionally and additively manufactured titanium components are often used in modular junctions. Consequently, understanding the tribocorrosion properties of new titanium alloys is critical to screening their performance as potential new biomaterials.

The relevance of wear properties for new AM Titanium biomaterials is directly related to their in vivo application. While crucial for articulating components like acetabular cups and modular taper junctions under cyclic loading, fretting performance is less important for mechanically inert, well-fixed devices. Data reported by the FDA on additively manufactured devices cleared from 2010 to 2020 reveals three trends relevant to this study [65]. First, 70 percent of all cleared AM devices (at least 357 devices) use titanium-based biomaterials. Second, 78 percent of devices were manufactured using laser powder bed fusion, the manufacturing method investigated in this paper. Third, spinal cages, where fretting corrosion is not a prominent damage mode, make up 46 percent of devices cleared via the 510 k pathway.

In addition to reinforcing the clinical relevance of this work, these trends reveal that orthopedic applications do not always involve interfaces where wear and fretting corrosion occur. To mitigate fracture within total knee replacement systems, AM tibial trays are produced through hybrid manufacturing. The keel (the portion of the device that interfaces with the patient’s bone) is printed onto a traditionally manufactured puck, leaving a visually distinct transition. Failure at this interface is hypothesized to initiate micromotion due to poor bony ingrowth [65]. Thus, even in instances where tribocorrosion is a concern, AM may allow device designers to pick and choose manufacturing processes and biomaterials to optimize desired properties and improve clinical performance.

Based on the wear and fretting results of this study and the fundamental corrosion properties we previously elucidated, we provide the following practical recommendations for using AM Ti-29Nb-21Zr as a biomaterial. First, despite comparatively decreased wear and fretting corrosion properties for the pre-alloyed Ti-29Nb-21Zr, it is unclear what impacts these would have in vivo, given the current applications for AM titanium alloys. The poor wear resistance of titanium and its alloys is well documented in the literature, and CoCrMo and ceramics like BIOLOX delta are favored for bearing surfaces like femoral heads in total hip replacement devices and femoral condyles in total knee replacement systems [53,66,67,68,69,70,71,72,73]. In other words, worse wear performance than Ti-6Al-4V does not preclude using AM Ti-29Nb-21Zr as an orthopedic alloy, given that other biomaterials are selected when wear is a major factor. Additionally, previous studies on AM Ti-29Nb-21Zr show good osseointegration (evidenced by 95% bone-implant contact in a sheep animal model), improved corrosion resistance in simulated inflammatory environments, and increased resistance to cathodic activation compared to Ti-6Al-4V [49,74,75]. In total, these studies, along with the wear properties reported here, support the use of Ti-29Nb-21Zr in vivo at bone-device interfaces, though further research is required.

## 5. Conclusions

In this study, we investigated the wear and fretting corrosion behavior of three new additively manufactured biomaterials, including pre-alloyed AM-29Nb-21Zr, admixed AM Ti-29Nb-21Zr, and Ti-6Al-4V nYSZ. We selected traditional and AM Ti-6Al-4V, two biomaterials actively used in vivo, for comparisons. Wear and fretting corrosion were quantified using a single diamond asperity (17 µm radius, 100 mN, 3 Hz, 100 cycles). While we found few differences between traditional and AM Ti-6Al-4V, we identified pre-alloyed AM Ti-29Nb-21Zr as the least resistant to wear and fretting corrosion (measured by wear track depth). We identified admixed AM Ti-29Nb-21Zr as the second least resistant to fretting corrosion. Additionally, compared with the Ti-6Al-4V-based biomaterials, AM Ti-29Nb-21Zr biomaterials generally exhibited less uniform wear and higher frictional forces during single-asperity fretting in air and solution. These results support our initial hypotheses where, based on previous corrosion studies of the conventionally manufactured Ti-13Nb-13Zr alloy, we expected decreased wear and fretting corrosion properties for the Ti-29Nb-21Zr biomaterials when compared with Ti-6Al-4V. Despite these decreased properties, Ti-29Nb-21Zr in its current as-built state may still be suitable for in vivo use at interfaces where bone ingrowth and fixation are prioritized (e.g., in the keel of tibial baseplates). Additionally, post-processing techniques, including diffusion hardening and surface coatings, may increase the wear and fretting corrosion properties of the Ti-29Nb-21Zr biomaterials, expanding their use cases in vivo. Future investigations will focus on characterizing the fatigue resistance of the new AM biomaterials, which is critical for in vivo success.

## Figures and Tables

**Table 1:** Printer parameters used to fabricate AM titanium alloys using L-PBF by an SLM 125 printer.

Power	Speed	Hatch Spacing	Layer Thickness	Laser Spot Diameter
200 W	700 mm/s	0.1 mm	30 µm	80 µm

**Table 2:** Average EDS analysis weight percent of each element measured in the fretting corrosion debris and wear track, with *p*-value comparison between biomaterial and elements from single factor ANOVA calculations, n = 5.

	Ti-6Al-4V Alloys				Ti-29Nb-21Zr Alloys		
	Traditional	AM	AM 1% nYSZ	*p*-Value	Admixed	Pre-Alloyed	*p*-Value
Ti	84.9 +/− 0.43	86.7 +/− 0.29	86.4 +/− 0.40	<0.001	45.2 +/− 1.16	52.5 +/− 0.41	<0.001
Al	6.34 +/− 0.11	5.88 +/− 0.08	5.66 +/− 0.09	<0.001	-	-	
V	3.44 +/− 0.05	3.54 +/− 0.05	3.66 +/− 0.05	<0.001	-	-	
Zr	0	0	0.68 +/− 0.04	<0.001	21.4 +/− 0.32	18.7 +/− 0.04	<0.001
Nb	-	-	-		28.7 +/− 1.71	22.3 +/− 0.30	<0.001
O (α = 0.025)	5.16 +/− 0.45	3.76 +/− 0.38	3.48 +/− 0.39	<0.001	4.32 +/− 0.73	6.12 +/− 0.31	0.001
P	0.12 +/− 0.04	0.16 +/− 0.05	0.10 +/− 0.00	0.10	0.30 +/− 0.00	0.40 +/− 0.00	<0.001

**Table 3:** Measured scratch depth after abrasion by a single micro-asperity, n = 5.

Wear Track Depth (µm)	Traditional	AM Ti6	AM Ti6 1%	AM TNX Admixed	AM TNZ Pre-Alloyed
Dry Fretting	2.90 +/− 0.32	2.51 +/− 0.52	2.03 +/− 0.12	2.80 +/− 0.65	4.67 +/− 0.55
Fretting Corrosion in PBS	2.09 +/− 0.59	1.16 +/− 0.79	1.42 +/− 0.64	3.61 +/− 0.96	5.78 +/− 0.83

**Table 4:** Average baseline current and average current above baseline during fretting (0 V vs. Ag Ag/Cl in PBS, n = 5).

	Traditional	AM Ti6	AM Ti6 1%	AM TNZ Admixed	AM TNZ Pre-Alloyed	*p*-Value
Baseline Current (µA)	0.10 +/− 0.07	1.19 +/− 0.50	0.10 +/− 0.02	0.82 +/− 0.40	0.72 +/− 0.26	<0.001
Fretting Current (µA)	0.37 +/− 0.05	0.34 +/− 0.05	0.36 +/− 0.05	0.42 +/− 0.04	0.58 +/− 0.07	<0.001

**Table 5:** Parameters used to calculate the oxide film properties and the oxide charge volume (C/cm^3^) of the five bioamterials tested. (Note: Weight % from EDS are average values calculated from Table 2 excluding O and P in the total %, n = 5).

Alloy	Element	wt% from EDS	*M_w_* (g/mol)	Molar %	Oxide	Oxide Density (g/cm^3^)	Oxide *M_w_* (g/mol)	Valence n	*Φ* (C/cm^3^)
Traditional Ti-6Al-4V	Ti	89.7	47.9	85.4	TiO_2_	4.23	79.9	4	
	Al	6.7	27	11.3	Al_2_O_3_	3.95	51	3	
	V	3.6	50.9	3.3	V_2_O_5_	3.36	91.0	5	
					Ti-6Al-4V	4.17	77.0	3.92	20,482
AM Ti-6Al-4V	Ti	90.2	47.9	86.3	TiO_2_	4.23	79.9	4	
	Al	6.1	27	10.4	Al_2_O_3_	3.95	51	3	
	V	3.7	50.9	3.3	V_2_O_5_	3.36	91.0	5	
					Ti-6Al-4V	4.17	77.3	3.93	20,471
AM Ti-6Al-4V w/1% nYSZ	Ti	89.6	47.9	86.2	TiO_2_	4.23	79.9	4	
	Al	5.9	28.0	10.0	Al_2_O_3_	3.95	51	3	
	V	3.8	50.9	3.4	V_2_O_5_	3.36	90.95	5	
	Zr	0.7	91.2	0.4	ZrO_2_	5.68	123.2	4	
					Ti-6Al-4VZr	4.18	77.54	3.93	20,450
AM Ti-29Nb-21Zr (admixed)	Ti	47.4	47.9	63.5	TiO_2_	4.23	79.9	4	
	Nb	30.1	92.9	20.8	Nb_2_O_5_	4.47	265.8	5	
	Zr	22.5	91.2	15.8	ZrO_2_	5.89	107.2	4	
					Ti-29Nb-21Zr	4.54	122.8	4.21	15,012
AM Ti-29Nb-21Zr (pre-alloyed)	Ti	51.2	47.9	66.9	TiO_2_	4.23	79.9	4	
	Nb	28.0	92.9	18.8	Nb_2_O_5_	4.47	265.8	5	
	Zr	20.9	91.2	14.3	ZrO_2_	5.89	107.2	4	
					Ti-29Nb-21Zr	4.51	118.8	4.19	15,346

## Data Availability

Data are available from the corresponding author (jlgilbe@clemson.edu) upon reasonable request.

## Abbreviations

AM	Additive Manufacturing
ANOVA	Analysis of Variance
BSE	Backscattered Electron
COF	Coefficient of Friction
DFD	Depth from Defocus
DI	Deionized
DOM	Digital Optical Microscope
DVRT	Differential Variable Reluctance Transducers
EDS	Energy Dispersive Spectroscopy
ELI	Extra Low Interstitial
F	Faraday’s Constant
FDA	Food and Drug Administration
FIG	Figure
L-PBF	Laser Powder Bed Fusion
Mw	Molecular Weight
n	Oxide Valence
nYSZ	Nano Yttria Stabilized ZrO_2_
OCP	Open Circuit Potential
PBS	Phosphate Buffered Saline
SE	Secondary Electron
SEM	Scanning Electron Microscope
SLM	Selective Laser Melting
Ti6	Ti-6Al-4V
Ti6 1%	Ti-6Al-4V with 1% nYSZ
TNX	Ti-29Nb-21Zr (admix)
TNZ	Ti-29Nb-21Zr (pre-alloyed)
wt%	Weight Percent
*ρ*	Oxide Density
*Φ*	Charge per Oxide Volume
